# Pupil dilation and constriction in the skate *Leucoraja erinacea* in a simulated natural light field

**DOI:** 10.1242/jeb.243221

**Published:** 2022-02-14

**Authors:** Lydia M. Mäthger, Michael J. Bok, Jan Liebich, Lucia Sicius, Dan-Eric Nilsson

**Affiliations:** ^1^ Marine Biological Laboratory, Bell Center, Woods Hole, MA 02543, USA; ^2^ Lund Vision Group, Department of Biology, University of Lund, 223 62 Lund, Sweden; ^3^ Westphalian Institute for Biomimetics, Westphalian University of Applied Sciences, Bocholt 43697, Germany; ^4^ Florida State University, Tallahassee, FL 32306, USA

**Keywords:** Environmental light field, ELF, Batoid, Elasmobranch, Vision

## Abstract

The skate *Leucoraja erinacea* has an elaborately shaped pupil, whose characteristics and functions have received little attention. The goal of our study was to investigate the pupil response in relation to natural ambient light intensities. First, we took a recently developed sensory–ecological approach, which gave us a tool for creating a controlled light environment for behavioural work: during a field survey, we collected a series of calibrated natural habitat images from the perspective of the skates' eyes. From these images, we derived a vertical illumination profile using custom-written software for quantification of the environmental light field (ELF). After collecting and analysing these natural light field data, we created an illumination set-up in the laboratory, which closely simulated the natural vertical light gradient that skates experience in the wild and tested the light responsiveness – in particular the extent of dilation – of the skate pupil to controlled changes in this simulated light field. Additionally, we measured pupillary dilation and constriction speeds. Our results confirm that the skate pupil changes from nearly circular under low light to a series of small triangular apertures under bright light. A linear regression analysis showed a trend towards smaller skates having a smaller dynamic range of pupil area (dilation versus constriction ratio around 4-fold), and larger skates showing larger ranges (around 10- to 20-fold). Dilation took longer than constriction (between 30 and 45 min for dilation; less than 20 min for constriction), and there was considerable individual variation in dilation/constriction time. We discuss our findings in terms of the visual ecology of *L. erinacea* and consider the importance of accurately simulating natural light fields in the laboratory.

## Teaser

**Summary:** A recently developed sensory-ecological approach to creating a controlled light environment in the laboratory yields robust behavioural data, providing a detailed account of the skates' pupil changes from near circular under low light to a series of small triangular apertures under bright light.

## INTRODUCTION

**Fig. 1.**
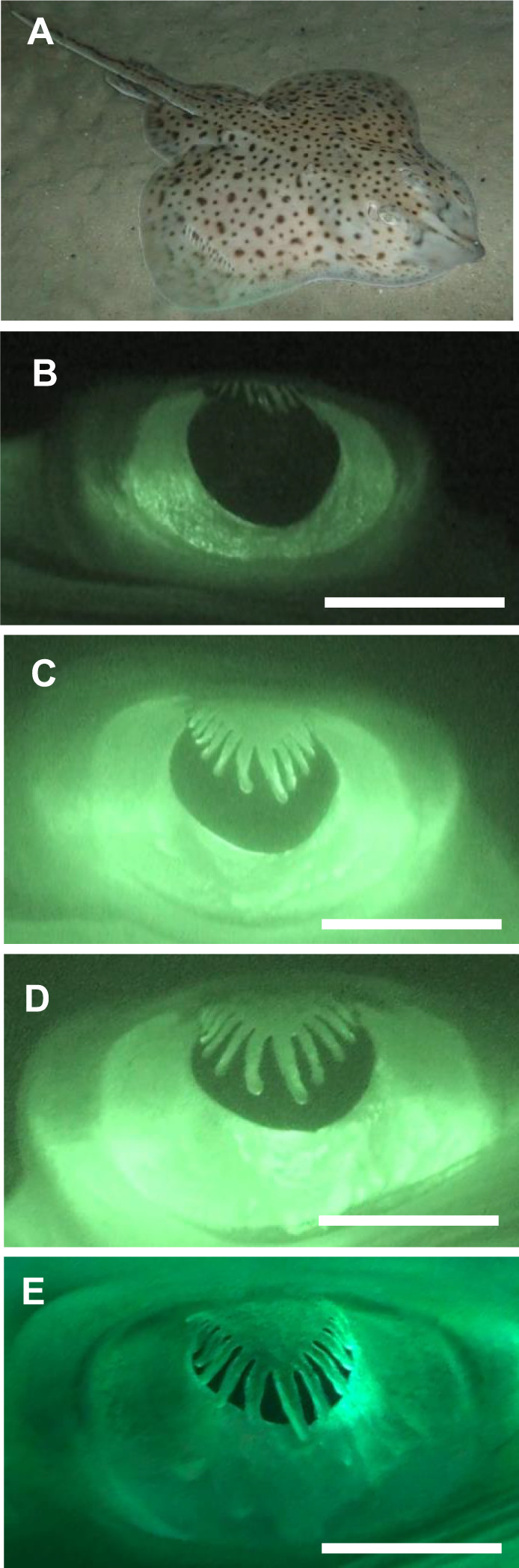
**The frill-like pupil of the little skate, *Leucoraja erinacea.*** (A) Whole-animal image, approximate length: 35 cm*.* (B–E) The light-responsive pupil changes from near circular under low light to an elaborate shape under bright light. (B) Lowest light setting, 0.00018 lx; (C) 0.12 lx; (D) 7 lx; (E) brightest light setting, 500 lx. Scale bar in B–E: 5 mm.

Pupils are found in the eyes of vertebrates and invertebrates, and most pupils generally respond to light by constricting. This limits the amount of light entering the eye (Denton, 1956; Wilcox and Barlow, 1975; Hammond and Mouat, 1985; Douglas, 2018; Land and Nilsson, 2012). In terms of visual function, we have a good understanding of circular, horizontal and vertical slit-shaped pupils (Nilsson et al., 2005; Malmström and Kröger, 2006; Lind et al., 2008; Land and Nilsson, 2012; Douglas, 2018) but there are several other pupil shapes that have not been studied very much at all, including the multiple-pinhole pupil found in geckos (Denton, 1956; Roth et al., 2009), the elaborate W-shaped pupil of cuttlefish *Sepia officinalis* (Muntz, 1977; Murphy and Howland, 1991; Douglas et al., 2005; Mäthger et al., 2013) and the frill-like pupil of the little skate, *Leucoraja erinacea*, which we studied here (Fig. 1; Kuchnow, 1971; Youn et al., 2019).

There is still much to learn about the full extent of pupil dilation and constriction in elasmobranchs. Earlier work suggests that, with some exceptions, mobile light-sensitive pupils are common in batoids (skates and rays), including *Raja* species. In selachians (sharks), pupils were generally assumed not to be mobile, although we now know that many shark species certainly have mobile pupils (Beer, 1894; Franz, 1906, 1931; Young, 1933; Walls, 1963; Kuchnow, 1971; Collin, 1988; Nicol, 1989; Douglas, 2018). However, systematic studies are lacking.

*Leucoraja erinacea* is an excellent batoid species to study these kinds of questions. It is easy to maintain in the laboratory and its comparatively small size makes experimental logistics more manageable. *Leucoraja erinacea* has a cathemeral lifestyle: it is irregularly active throughout night and day. However, some evidence looking at metabolic rate at different times of night/day suggests that it is slightly more active during crepuscular and nocturnal times of day (Hove and Moss, 1997). Other skate species follow similar patterns. Activity has been recorded day and night, with a slight crepuscular/nocturnal increase (Wearmouth and Sims, 2009). As has been shown for several other elasmobranch species, *L. erinacea* has monochromatic vision. Its retina has been reported to contain only rods with an absorbance peak at 500 nm (Dowling and Ripps, 1970; Green and Siegel, 1975).

More recently, while testing the visual field characteristics of four batoid elasmobranch species, McComb and Kajiura (2008) reported a wide-open pupil during dark adaptation but did not further test pupil light responsiveness or dilation/constriction rates. Similarly, pupil dilation and visual field were studied in dogfish (Kajiura, 2010) but no details regarding the full extent of dilation or constriction were given. McComb and Kajiura (2008) and Kajiura (2010) anaesthetized their animals using MS222 to facilitate handling and prevent animals from moving during experimentation. Although the authors discuss that anaesthesia may have negatively influenced the natural light response of the batoids' eyes, they deemed it unlikely that the anaesthetic dramatically affected the response. In contrast, Hove and Moss (1997) report that MS222 does indeed disrupt the metabolism associated with vision. Unfortunately, thorough studies are lacking, but we can say with certainty that anaesthesia negatively impacts behavioural and physiological studies, including visually related physiological responses (e.g. Charng et al., 2013).

Given the likelihood that drugs affect vision, the pupillary response should ideally be studied in unmedicated animals. Provided that skates were handled carefully, and given size-appropriate tanks and sufficient acclimation time, we found that skates voluntarily settled in our experimental tanks and remained calm for experimental trials to be conducted without anaesthesia.

In live animal experimentation concerned with physiology and behaviour, researchers are generally aware of the potential negative effects of experimental/laboratory techniques on an animal's response. Studies of animal behaviour and/or physiological responses are difficult and time consuming, requiring intense planning, testing and trouble shooting of experimental methods, so that results are accurate. Still, even the most carefully conducted experiment may leave doubt whether a given method negatively affects the very response one intends to quantify. One area that has received scant attention is the ambient lighting in which animals are kept, and in which experiments are conducted. Traditionally, laboratories are equipped with white-light fluorescent, incandescent or LED lighting, paying little attention to what an animal's natural light field may be. Similarly, experiments are usually designed around the recording cameras: the brighter the light, the better the recording quality. Luckily, with the advancement of technology, more and more low-light sensitive and night-vision cameras have become available, finally making high-wattage photographic lights for animal behavioural work obsolete. In the present study, we have taken a new sensory–ecological approach to performing a physiological experiment in a freely behaving animal. Importantly, this new tool can be applied to any animal behavioural experiment. We conducted a field survey to obtain data on the natural light field of skates (*L. erinacea*), and created an ambient light field using recently developed software (environmental light field, ELF; Nilsson and Smolka, 2021). For our laboratory experiments, we then used these field data to re-create, as best we could, a laboratory light field that closely simulated the natural vertical light gradient that skates experience in the wild. The advantage of using a tool of this kind is far reaching in that it allows us to correlate real-world situations at different times of day and different depths with the animal's pupil state.

Using this improved sensory–ecological approach, the goal of our study was to document the pupil dynamics of unrestrained and unmedicated *L. erinacea*, under naturalistic lighting conditions. In particular, we wanted to quantify visually important parameters such as the extent of dilation and constriction and the speeds involved.

## MATERIALS AND METHODS

### Animals

The little skate *Leucoraja erinacea* (Mitchill 1825) (Fig. 1A) is common off the coast of Woods Hole, MA, USA. Animals are regularly collected, as well as raised in captivity at the Marine Resources Center of the Marine Biological Laboratory (MBL). All animals used were wild-caught (within less than a year). Skates were kept at 12–15°C in a large holding tank (1.2 m×3.6 m; 40 cm height) surrounded by large windows. During the weeks of these experiments, the animals' dark cycle lasted from approximately 20:00 h to approximately 05:00 h. Skates were identified by sex and length, as well as individual physical characteristics (e.g. identifying scars, lines or spots, etc.), and fed 5 times a week (variation of squid, butterfish and capelin). All animals were cared for and experiments were conducted in accordance with the regulations of the MBL Institutional Animal Care and Use Committees.

### Natural ELF data

**Fig. 2.**
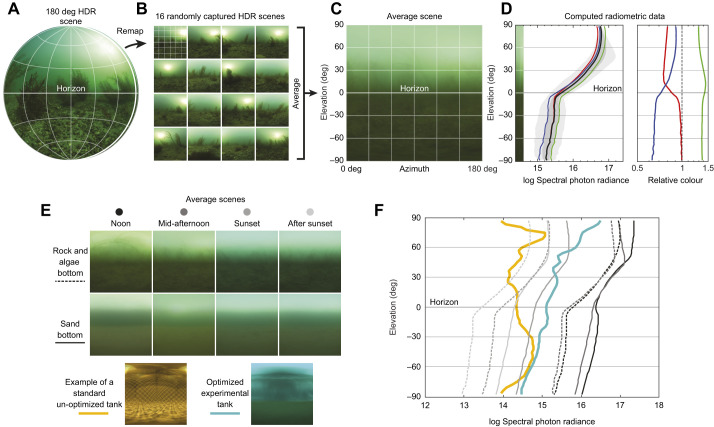
**Environmental light field (ELF) analysis of natural *L. erinacea* habitats and laboratory set-ups.** The ELF method uses 180 deg high dynamic range (HDR) images taken in a specific natural habitat levelled to the horizon (A). Multiple individual scenes are captured at random orientations throughout the habitat. They are remapped (B) and averaged (C) to create a mean profile of the vertical light gradient. From this, radiometric data (spectral photon radiance in photons m^–2^ s^−1^ sr^−1^ nm^−1^) are computed along the vertical light gradient (D). We used this method to analyse the natural habitats of skates at various times of the day and aid in the design of an optimized experimental tank (E,F). An example of an un-optimized tank set-up using a black and white checkerboard substrate is given. Line formats (solid or dotted) and shades (greys, yellow, teal) of the radiometric plots in F refer to the averaged scenes in E as indicated. Full ELF analysis plots are available in Fig. S1.

Using SCUBA, we collected light field data in the natural habitats of *L. erinacea* from 18 to 20 October 2016 according to the ELF method described by Nilsson and Smolka (2021)*.* The data collecting apparatus consisted of a SLR camera (Nikon D810), equipped with a Sigma 8 mm f/3.5 EX DG circular fisheye lens, placed in an underwater housing that was custom-made to allow the full 180 deg field of view of the fisheye lens to be used (The Sexton Corporation, Salem, OR, USA). We collected data at two typical local skate habitats (all approximately 3–5 m depth): site 1 was a rubble, sand and sargassum habitat off Stony Beach in Woods Hole, MA, USA (41.516537, −70.659976); site 2 was a sandy habitat off Nobska Beach in Woods Hole, MA, USA (41.529946, −70.674377). Data were collected at four times throughout the day: at solar noon, mid-afternoon, and just before and after sunset. For all datasets, the weather was clear and sunny with few clouds. Images were collected with the camera housing resting on the substrate. Between 12 and 21 scenes were imaged at each site depending on the structural visual complexity of the sites, each with the camera levelled to the horizon. Sites without algae or rocks required fewer scene images to create a homogenous average image. Each scene was imaged at randomly selected horizontal directions and utilized three bracketed exposures each to produce high dynamic range images (Fig. 2A,B). The scenes imaged at each site were then analysed according to the ELF method (Nilsson and Smolka, 2021). Briefly, average images were generated for each set of scenes (Fig. 2C). From these averaged images, calibrated spectral photon radiance information was extracted along the vertical axis from straight up (90 deg), through the horizon (0 deg), to straight down (−90 deg) (Fig. 2D). These data gave us a powerful tool for re-creating a natural light field in the laboratory.

### Experimental set-up

**Fig. 3.**
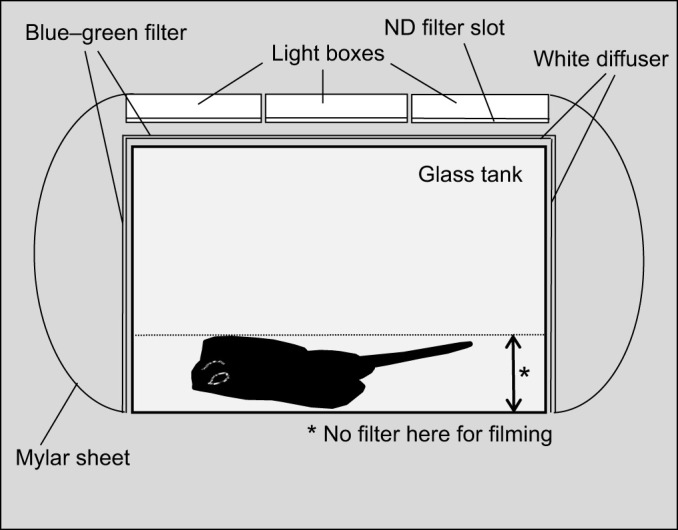
**Experimental set-up.** The set-up that was designed and built to simulate the natural light field of *L. erinacea*. An ice fishing tent (indicated by the dark grey box surrounding the tank) was used to house a glass tank, surrounded by blue–green and white diffuser filters. Reflective Mylar material was placed along the sides of the tank. At the front of the tank, the filters were lifted to provide a small window for filming. See Materials and Methods for more details.

Our experimental set-up (Fig. 3) consisted of a glass tank that was placed inside an improvised dark room constructed from a black ice fishing tent surrounded by black plastic sheeting to block out all exterior light. The glass tank dimensions were: 61 cm length, 61 cm width, 46 cm height. To give skates a more natural bottom, we lowered a Plexiglas sheet onto the floor, which consisted of glued-on natural sand and small pebbles (glued on using silicone adhesive). The overhead light source consisted of three side-by-side custom-made light boxes, lined with LE Flexible Strip, SMD 2835 Daylight White LEDs to provide even illumination across the entire tank. The light boxes were constructed in such a way that neutral density filters (a combination of 0.15 ND and 0.6 ND, Lee Filters) could be inserted to decrease the light intensity to the desired level. The outside of the experimental tank was lined with a white diffuser (Lee Filters half white diffusion) as well as a blue–green filter (Roscolux #374 sea green) (note: the ‘outside’ refers to all four tank sides and a tight-fitting lid that was placed on top of the tank during experiments to completely ‘seal’ the experimental tank, except for a small opening for filming). Around the outside of the experimental tank, secured to the inside of the ice fishing tent, we placed sheets of 2 mm reflective Mylar (Virtual Sun Hydroponics, Inc.), which effectively channelled light downwards and increased the relative light intensity coming into the experimental tank from the sides. Our goal was to design the experimental light field to match the average natural light field characterized using the ELF method as closely as possible (Fig. 2E,F). This took some time to achieve, and filters, light sources and tank had to be adjusted multiple times. During the set-up phase, an Extech EasyView 30 light meter (irradiance, measured in lux) was used to guide us in the selection and placement of filters, locations of light sources, and lighting intensities. This type of light meter is convenient because light data are immediately available and progress is not delayed by the time-consuming computation that is often required for obtaining radiometric light data. However, as lux meters are based on the human visual system, they have drawbacks when used in the context of animal vision. Despite this, the use of these instruments may be justified when working with monochromatic animals (e.g. skates) because lux meters are most sensitive in the green part of the spectrum. In addition to using a lux meter, we also measured the radiance inside the tank at different vertical angles using an Ocean Optics QE65000 spectrometer (Ocean Optics, Dunedin, FL, USA) and a 400 µm fibre (QP400-2-UV/VIS) with an attached Gershun tube (Gershun tube kit, Ocean Optics). The fibre was attached to a goniometer, which allowed measurements to be taken in 10 deg steps. A calibration light source (LS-1-CAL, Ocean Optics) was used to convert data into absolute radiance. Radiance data were converted into photons m^−2^ s^−1^ sr^−1^ nm^−1^. We then used the same underwater camera system and ELF method described above to analyse the light field of the experimental set-up. Final set-up adjustments were made to obtain an experimental light field that fell well into the types of light fields measured in the natural habitats of skates (Fig. 2B).

### Experimental procedure

#### Experiment 1: speed of dilation/constriction of skate pupil

For this experiment, 11 skates (body length 33–38 cm) were used. All experiments were conducted between 08:00 h and 17:00 h (their ‘dark cycle’ lasted from approximately 20:00 h to 05:00 h). Skates were placed in the experimental tank at full light intensity (500 lx, measured with the Extech EasyView 30 light meter, placed on the bottom of the tank at the level of the skates' eyes), and given approximately 30 min acclimation time. After acclimation, the lights were turned off and the skate's pupil dilation was filmed for 10–20 s every 3–5 min. When no further changes in pupil dilation were observed, the light was turned on, and pupil constriction was filmed for 10–20 s every 3–5 min until no further changes in pupil constriction were observed. It was important to allow sufficient time for the skate pupil to fully adjust. Skates were given approximately 5–10 min after the experimenter had decided that full constriction/dilation had been achieved to ensure there were no further subtle pupillary changes.

All video recording was done using a Sony HDR-XR550VEB video camera, which was placed perpendicular to the skate's eye, so that the entire pupillary area could be monitored. During dark adaptation, the night-shot setting of the camera was used.

Skates generally settled quickly and positioned themselves in such a way that one eye was perpendicular to the camera; however, on the occasion that a skate shifted, we were able to move it gently to position it perpendicular to the camera. Usually, the skates did not react to being moved and remained settled. Occasionally, if a skate did react by swimming around, it was given more acclimation time until it was settled. The skates that were available to us had a range of temperaments, and when we initially selected animals for these trials, we avoided selecting animals that would not settle in the experimental tank within an hour.

##### Analysis

From each video segment, a single image, showing the eye in a position perpendicular to the camera, was extracted. For each animal, we had 11–26 images for the entire constriction/dilation speed trial. These images were then used to measure the effective pupil area using ImageJ (NIH). We used the polygon selection tool to trace the pupil margins, so that the effective pupil area could be measured.

#### Experiment 2: response of skate pupil to changes in intensity

Using neutral density filters (0.6 ND and 0.15 ND), we created 10 light intensities. The highest light intensity, without any neutral density filters, was 500 lx (less than 5% variation across the experimental tank, verified by Extech EasyView 30 light meter). The next lower light intensity was obtained by adding one 0.6 ND filter, which reduced the light intensity to 125 lx. The next lower light intensity was obtained by adding a second 0.6 ND filter, which reduced the light intensity to 31.25 lx, and so on. The lowest light intensity, 0.00018 lx, was obtained by adding eight 0.6 ND filters and one 0.15 ND filter.

Before experiments started, all neutral density filters were placed in front of the light sources, resulting in the lowest light intensity (0.00018 lx). Filters were progressively removed to proceed to the next brighter light field. As before, the video camera was placed perpendicular to the skate's eye. The night-shot setting of the camera was used for all but the brightest light intensity setting.

Ten skates (body length 33–38 cm) were used for this experiment (these were the same skates as used for experiment 1 but we lost one skate; in the results and figures, these skates are referred to as group 1). Additionally, we found that a linear regression analysis was useful in interpreting our data, so data from a pilot experiment were added, referred to as group 2. Skates in group 1 were overall larger and slightly older than the skates in group 2, although the sizes overlapped to some degree. This added six smaller skates (body length 25–33 cm) to the dataset. All methods were identical to those described above; the only difference was the size of the experimental tank (50 cm length, 40 cm width, 30 cm height) and a slightly higher light intensity at the brighter light setting (at the highest setting, a difference between 500 lx versus 700 lx), which caused no noticeable difference in pupil constriction (note, the reason for the slight discrepancy in the set-up was that larger animals needed a larger tank, which also meant a larger light source had to be employed).

Each skate was taken out of its holding tank and placed in the experimental tank, where it was given approximately 30 min adaptation time. During this time, the pupil dilation process was monitored, and experiments began when full dilation had been reached and no further dilation movements were detected. When full dilation was reached, the skate was filmed for approximately 1 min. We then removed neutral density filters to proceed to the next brighter light setting and allowed another 30 min adaptation, during which the pupil dilation was recorded as described before. After 30 min, the skate was again video-recorded for approximately 1 min. This procedure was repeated for each light intensity.

##### Analysis

As before, only images showing the eye in a position perpendicular to the camera were selected. From the 1 min video clips taken at each light intensity, we extracted 4 images at intervals of approximately 5 s. The pupil margin and effective pupil area were determined in the same way as described for experiment 1. Data obtained for the four images were averaged. In the Results, we show dilation/constriction of all 10 skates. Additionally, to test the relationship between eye size and dilation/constriction state, we used Microsoft Excel to perform a linear regression analysis.

## RESULTS

### ELF analysis of skate habitats

We performed ELF analysis at two natural study sites of *L. erinacea* to develop the optimal light conditions for our experimental use. In Fig. 2, we show the steps that led to our final dataset. Individual images were first re-mapped to correct for the camera's equisolid image projection. The resulting images were square, consisting of a new array of pixels with equal angular span in the vertical and horizontal fields of view. Using these images, pixels were averaged (Fig. 2C), revealing a vertical gradient profile, from which radiometric data were computed (Fig. 2D). As every natural light field undergoes circadian changes owing to the position of the sun/moon, cloud cover, etc., we collected data at different times of day (Fig. 2E,F; Fig. S1). We found that vertical intensity gradients of light in both natural habitats presented a similar profile that decreased in intensity uniformly from noon to sunset, with only some subtle angular changes. Snell's window, which occupies angles of elevation between 41.5 and 90 deg, provided the brightest light (above 17 log_10_ spectral photon radiance units at noon in our sandy bottom site). Beyond Snell's window, the intensity decreased sharply towards the horizon. Below the horizon, the intensity continued to decrease gradually, which is partially caused by the camera's shadow. Green and blue wavelengths dominated above the horizon but decreased below the horizon, while red wavelengths increased slightly (Fig. 2D; Fig. S1).

With these data in mind, we set out to create an artificial light field that simulated the natural light field as closely as possible. We made use of a variety of filters (see Materials and Methods) and repeatedly adjusted these, as well as the overhead light sources, until we succeeded in obtaining a light field with an intensity and relative colour gradient similar to the ELF averages (teal line in Fig. 2F; Fig. S1). Importantly, it became obvious that the use of artificial backgrounds, which are often employed in behavioural experimentation because of the ease with which spatial frequency and intensity can be modulated, created an artificial light field that did not resemble the natural light field at all (yellow line in Fig. 2E,F; Fig. S1).

### Speed of dilation/constriction of skate pupil

**Fig. 4.**
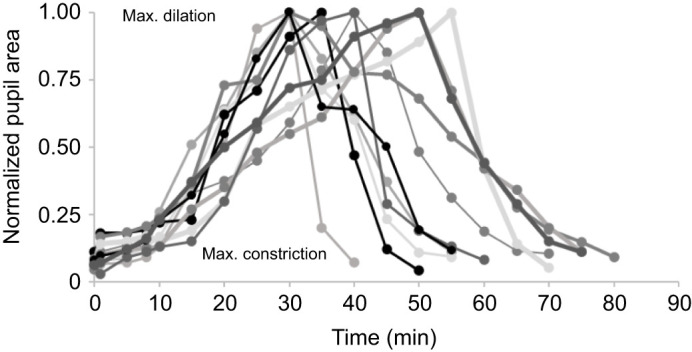
**Dilation and constriction speeds for 11 skates (*L. erinacea*).** For comparative reasons, data were normalized; i.e. each skate's maximum dilation area was given a value of 1. In the initial 8–10 min, little dilation was observed. Most dilation occurred after 10 min following the onset of dark adaptation. There was considerable variation in dilation time between skates. Constriction was generally faster; it was achieved in under 20 min. At time 0, lights were turned off. Lights were turned on at different times for each skate, determined by when maximum dilation was achieved.

Dilation took longer than constriction, and there was individual variation in the time it took for the skates' pupils to fully dilate and constrict (Fig. 4). For all skates, dilation took from 30 min to over 50 min; constriction was usually achieved in under 25 min (Fig. 4, Table 1). Interestingly, in the first 8–10 min following the onset of dark adaptation (i.e. after lights were turned off), there was very little dilation. Most of the dilation occurred after approximately 10 min, and approximately 5 min before full dilation was achieved. Conversely, during light adaptation (i.e. after lights were turned on), most constriction occurred in the first 10 min; within the final minutes, constriction was slower. As Table 1 shows, pupil constriction area was relatively similar across all animals (mean±s.d. 1.3±0.37 mm^2^, range 0.8–1.7 mm^2^). Dilation area varied more (mean±s.d. 16.3±4.56 mm^2^, or 17.4±2.88 mm^2^ for the three skates that were tested a second time).

**Table 1.**
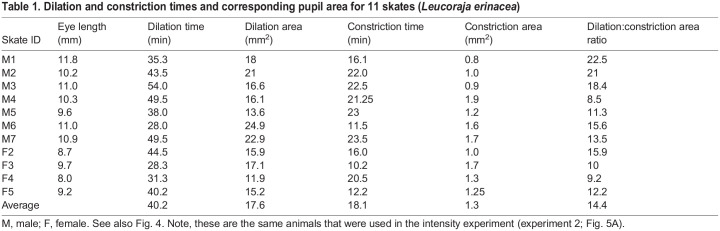
Dilation and constriction times and corresponding pupil area for 11 skates (*Leucoraja erinacea*)

### Response of skate pupil to changes in intensity

**Fig. 5.**
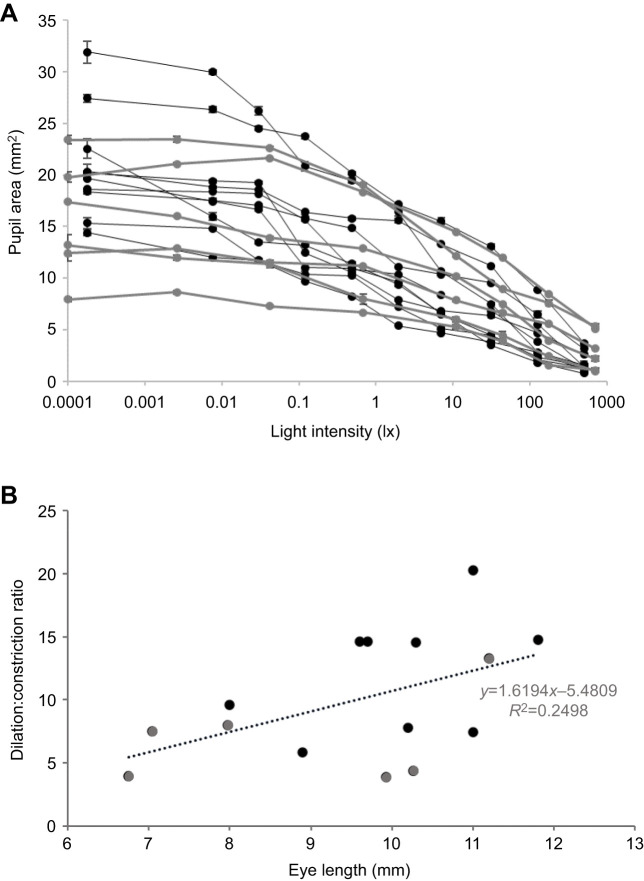
**Changes in skate pupil area under different light intensities.** (A) Response of 10 larger/older skates (black lines; group 1, 33–38 cm body length) and six smaller/younger skates (grey lines; group 2, 25–33 cm body length) to 10 light settings inside an experimental set-up that simulated a natural underwater light environment. The *x*-axis is presented on a log scale (note, lux values are given for ease of replicating this set-up; the equivalent radiometric value at maximum light intensity, measured with Ocean Optics equipment, was around 2e+17 photons m^−2^ s^−1^ sr^−1^ nm^−1^). (B) The ratio of dilation to constriction (maximum:minimum dilation) for skates of different sizes from group 1 (black circles) and group 2 (grey circles). A linear regression analysis shows that skates with smaller eyes (i.e. younger skates) achieved a smaller dilation:constriction range compared with skates with larger eyes (i.e. older skates); *n*=15. One outlier (with a value of 1.5 times the interquartile range) was removed from this analysis. The regression equation was not statistically significant (*F*_1,13_=4.33, *P*=0.057); however, there was a clear trend.

There was individual variation in the degree to which the pupil constricted in response to light but all skates followed the same pattern: during the darkest light setting, the pupil took on an almost circular shape; as light intensity increased, the pupil became more elaborate: the frilled dorsal portion lengthened from dorsal to ventral, resulting in a crescent shape with frilled elongations dropping down from the top of the eye. At the highest light intensity, the resulting series of small triangular apertures (e.g. Fig. 1E) caused a drastic reduction in effective pupil area (Fig. 5A). Notably, the overall pupil area (dilated and constricted) of smaller (and younger) skates (mostly in group 2) was smaller than that of larger (and older) skates (mostly in group 1). To determine the extent of the pupil area change (dilation:constriction ratio), we combined the data shown in Fig. 5A in a linear regression analysis (see Fig. 5B). Looking at this combined dataset, we found that skates with smaller eyes (i.e. younger skates) achieved a smaller dilation:constriction range (i.e. the area change from completely dilated to completely constricted was lower) compared with that of skates with larger eyes (i.e. older skates). The constriction state, however, was similar across all animals, irrespective of size: When constricted, the mean (±s.d.) pupil area was 2.36±1.44 mm^2^. However, dilation state varied dramatically: when fully dilated, the average pupil area was 18.69±5.96 mm^2^ (note the much higher standard deviation, indicating a larger variation). This shows clearly that, as the eye grows, skates are able to dilate their pupils more. The linear regression analysis that tested the relationship between eye size (here, we used eye length as an indicator of eye size) and dilation:constriction ratio showed that dilation:constriction ratios were smaller in smaller skates and greater in larger skates. Although not statistically significant, the regression equation (*F*_1,13_=4.33, *P*=0.057, with an *R*^2^ of 0.2498; see Fig. 5B) demonstrated a trend.

## DISCUSSION

It is important to provide experimental settings that allow natural behaviours to ensue as much as possible. Field studies are challenging owing to a huge array of variables that are impossible to control. This often makes the laboratory the only place where natural behaviours can be quantified. When conducting behavioural experiments in the laboratory, it is important to consider the effects of the laboratory environment (e.g. artificial tanks, lighting, etc.) on the outcome of the behaviours that are under investigation. Conventionally, researchers choose methods that optimize and maximize data collection, e.g. sufficient high-wattage lighting for clear video recording; small experimental chambers, harnesses or anaesthesia to prevent unwanted movement, etc. It is difficult to determine the potential negative impact of such methods on the behaviour that is being quantified, and is often impossible to design control experiments to test these potential effects. Here, we show how a comparatively simple method introduced by Nilsson and Smolka (2021) yields data on natural light fields that can subsequently guide laboratory experimental methods much better than lux meters or spectrometers, which do not take into account the structures and objects that a given visual scene contains. While the light data obtained by lux meters/spectrometers can be useful in guiding an experimental design, ultimately, we found that the ELF method provided us with the most accurate tool for simulating a natural light field in the laboratory.

Interestingly, while the skate *L. erinacea* has been a study organism for a variety of research topics (visual and non-visual) (Dowling and Ripps, 1970, 1972, 1990; Koester and Spirito, 2003; Gillis et al., 2013), the extent and rate of pupillary movement have not been documented. In our study, we aimed to give skates a laboratory setting that was as natural as possible, so that we could make inferences about their natural pupillary responses to light.

Not all fish have light-sensitive pupils. Although pupil mobility has been documented in several teleost species, the pupils of most teleost fish do not vary in response to ambient light changes (Nilsson, 1980; Somiya, 1987; Fujimoto et al., 1995; Douglas, 2018; Douglas et al., 1998, 2002). Within the elasmobranchs, there appears to be a similarly diverse distribution of mobile pupils (Beer, 1894; Franz, 1906, 1931; Young, 1933; Walls, 1963; Kuchnow, 1971; Collin, 1988; Nicol, 1989; Douglas, 2018). Kuchnow (1971) performed an investigation of a range of elasmobranch species, testing members of three selachian orders (Carcharhiniformes, Heterodontiformes, Squaliformes) and two batoid orders (Myliobatiformes, Rajiformes), and found mobile pupils in all but the deep-sea species (two spp. from the Squaliformes order). In the species Kuchnow (1971) worked on, the dilation/constriction ratios ranged from 1.2:1 to 50:1, demonstrating clearly the wide variety of pupil area changes in this animal group (see Table 1 for dilation:constriction ratios in *L. erinacea*). Pupil dilation:constriction speed also varied: some dilated and constricted in a matter of a minute or two, whereas others took half an hour or longer. The low sample size in Kuchnow's (1971) study makes it difficult to gain a more solid understanding of dilation and constriction extent and speed. Another important aspect to consider is that low-light video equipment has improved enormously since the 1970s and it is likely that Kuchnow (1971) would not have been able to track head movements as carefully as is possible now. Thus, the measured pupil areas in Kuchnow's (1971) work may not be as exact as necessary to fully characterize these pupil movements. Some of Kuchnow's (1971) data were obtained during cruises, making steady experimental conditions difficult.

Because of the large sizes of many elasmobranch species, studying their pupil light responses is a challenge. Furthermore, pelagic species (e.g. selachians) are even more difficult to study than benthic batoids, and it is therefore not surprising that the current literature on pupil mobility in elasmobranchs is so patchy. Clearly, there has been a need for more work in this area, particularly with regard to the diversity of body and head shapes, as well as lifestyles of elasmobranchs.

Our results undoubtedly show that light intensity causes significant changes in the pupil area of *L. erinacea*, with the pupil going from nearly circular to an elaborate crescent shape with multiple triangular apertures. As we can see in Fig. 5B, this change in area from constricted to dilated varied from nearly 4-fold in smaller (and younger) animals to 10- and up to 20-fold in larger (and older) animals (see also Table 1). While not statistically significant in our study, a trend was seen. A larger sample size may have added statistical significance to this finding.

An important consideration of this experiment is to ensure that skates are given sufficient time for full dilation/constriction to occur. Each animal has a different response time and data inaccuracies can easily occur. In the experiments that are shown in Table 1, several animals appeared initially to have completed the full constriction/dilation cycle but upon data analysis we suspected that the experimenter had determined too early that full dilation/constriction had been reached. After repeating the trials for these animals, it became apparent that these individuals did indeed need more time (an additional 5–10 min) until full dilation/constriction was achieved. Why individual skates (and possibly even the same individual) may have different dilation and constriction times remains to be investigated. This result could be due to numerous factors, such as circadian rhythm, seasonal aspects (e.g. breeding, etc.), hunger status, etc., none of which were controlled for in this study. However, it can be stated with confidence that we can rule out unintended light intensity variations, which were carefully controlled for with our sensory–ecological approach.

Why pupil dilation and constriction are so slow in skates is an interesting question that remains to be fully explored. Certainly, the light intensities that these cathmeral (day and night active) skates are exposed to on a daily basis do not change as fast or as drastically as they do for some other aquatic species, or compared with terrestrial organisms, suggesting that fast dilation and constriction are really not needed. Sun exposure, cloud coverage, etc., have a considerable impact on light intensity, both on land and underwater, and while underwater light fields may not be subject to such changes as fast or as drastically as terrestrial light fields, these changes are certainly noticeable, particularly at shallow depths (Jerlov, 1976; Cronin and Shashar, 2001). However, the most dramatic light intensity changes are associated with an animal's lifestyle. Moving in and out of crevices, caves and other forms of light-reducing/eliminating coverage can present light intensity differences of several orders of magnitude. Skates do not have this kind of an active lifestyle. They are benthic sit-and-wait predators that prefer open sandy sea floors, near shore to depths of about 90 m (Robins and Ray, 1986). From that perspective, skates are unlikely to experience light intensity changes beyond the natural circadian changes and those resulting from clouds covering the sun.

Interestingly, at the morphological level, the skate retina contains only rods, which, in general, are more light sensitive than cones but they do not function during photopic light intensities. Skate rods have solved this problem by taking on cone-like physiological properties under bright light (Dowling and Ripps, 1970), which supports the skate's cathemeral lifestyle. Additionally, their temporal response times are slower compared with those of other species (Dowling, 2012); thus, to support a cathemeral existence, skates may not need a fast-acting pupil.

Surprisingly, we found that larger skates had greater pupil dilation:constriction ratios than younger animals. The pupil area at constriction was relatively similar across all skate sizes/ages; however, the dilated pupil area was greater in larger (i.e. older) skates. To the best of our knowledge, this has so far not been observed in any animal. Interestingly, in humans, pupil size changes with age: it is smaller in younger children, maximum at an age of around 20, but then decreases with age to be smaller than that of a young child, which in turn reduces the amount of light that reaches the retina (Weale, 1961; Winn et al., 1994). While we used skates of different size ranges in our study, we cannot make any inferences regarding age beyond suggesting that the smaller animals were likely younger, based on their husbandry/collection history. A carefully controlled study on different skate age groups, including hatchlings, juveniles and late adult stages, would be extremely interesting. The reason for the variation in the dilation:constriction ratios remains to be investigated. There are obvious visual advantages to a larger pupil size, such as increased sensitivity in low light, although a large pupil size comes at a potential cost in that it may contribute to optical aberrations, which result in poorer resolution. Future studies should look at the interactions between lifestyle and ontogeny (retinal development, as well as overall body design) to determine the reason for a larger dilation:constriction ratio in older skates.

It is unclear why the speed of dilation and constriction in skates varied so much between individuals. In cephalopods, a similar variation has been reported (Douglas et al., 2005; Soto et al., 2020), although the cephalopod pupillary light reflex is much faster than that in skates, and also responds to stimuli other than light (e.g. during accommodation for hunting, as well as mating; Muntz, 1977; Messenger, 1981), which can confound light-induced dilation and constriction speeds. In vertebrates, there are several species for which non-light-induced pupillary movements have been described. For example, in humans, pupil dilation is affected by many factors, including hormones, emotions and behaviours, as well as visual and auditory perception, and it has been shown that the human pupillary light response is neither fully reflexive nor under complete voluntary control (Lowenfeld, 1993; Barbur et al., 2002; Einhäuser et al., 2007; Mathôt et al., 2013). In birds, pupil movements have been called ‘eye pinning’, and it has been suggested that they are used as a form of communication (Gregory and Hopkins, 1974). All of these non-light-induced pupillary changes are fast and subtle, and reflect a particular short-term physiological condition or have a particular short-term optical function. In our study, we did not test any factors, other than light intensity, that influence pupil size in skates. Furthermore, as pupillary movement is so slow in skates, it seems doubtful that skates have the ability to vary their pupil dilation/constriction to the degree that is reported in cephalopod or human subjects. It therefore seems unlikely that the variation in individual dilation/constriction speed reported here is due to non-light-related factors. However, in a separate study (Youn et al., 2019), we found that the skate pupil also changes in response to the spatial frequency of the background, so skates certainly appear to have the ability to change pupil dilation/constriction irrespective of light intensity, although at a much slower rate.

Another aspect that adds complexity to the pupillary response in skates is the connection between the two eyes. In many animals, the pupil response of each eye is independent of the other eye [e.g. some teleost fish (Steinach, 1890; Young, 1931; Nilsson, 1980); some sharks (Franz, 1931; von Studnitz, 1933; Kuchnow, 1971); some amphibians, reptiles and birds (Denton, 1956; Werner, 1972; Henning et al., 1991; Schaeffel and Wagner, 1992)], but there are also many species that have consensual response; that is, one eye responds when the other eye is stimulated [e.g. some teleosts (Douglas et al., 1998); some rays (Bateson, 1890; Franz, 1931; von Studnitz, 1933); some amphibians (von Campenhausen, 1963); and probably most mammals, including humans (Lowenfeld, 1993)]. According to Bateson (1890) and Franz (1931), skates (including *L. erinacea*) have a consensual pupillary reflex. Therefore, when studying animals that have a consensual pupillary light reflex, this is an added challenge that should be considered, and would be interesting to investigate in these skates.

Although beyond the scope of this study, the functional reasons for the elaborate pupil shape found in many skates deserves some comment. The circular shape of the fully dilated pupil makes perfect sense for maximizing visual sensitivity in dim light because it exploits the full aperture of the lens over the full visual field. Certainly, large eyes and circular pupils are common in nocturnal animals (Land and Nilsson, 2012). The adaptive value of the constricted pupil with multiple triangular pinholes is more obscure. The row of small apertures will generate specific blur patterns for objects that are out of focus because they are too close or too far away to be sharply imaged on the retina. This may be used as a mechanism for distance estimation. Another possibility is that wave-optics phenomena in the row of minute apertures reduces diffraction blur to improve the detection of high spatial frequencies in bright light. It is also possible that the elaborate pupil contributes to even out retinal illumination, as was demonstrated for the elaborate pupil of cuttlefish (Mäthger et al., 2013). A role in camouflaging conspicuous eyes can of course not be ruled out. It is likely that several of these functional reasons have contributed to the evolution of elaborate pupils in skates.

## Supplementary Material

10.1242/jexbio.243221_sup1 Supplementary information
