# Supplementary material for: Pupil dilation and constriction in the skate Leucoraja erinacea in a simulated natural light field
Source: J Exp Biol. 2022 Feb 14;225(4):jeb243221. doi: 10.1242/jeb.243221 (PMC10215813; doi:10.1242/jeb.243221)
Supplement: Supplementary information [file jexbio-225-243221-s1.pdf]

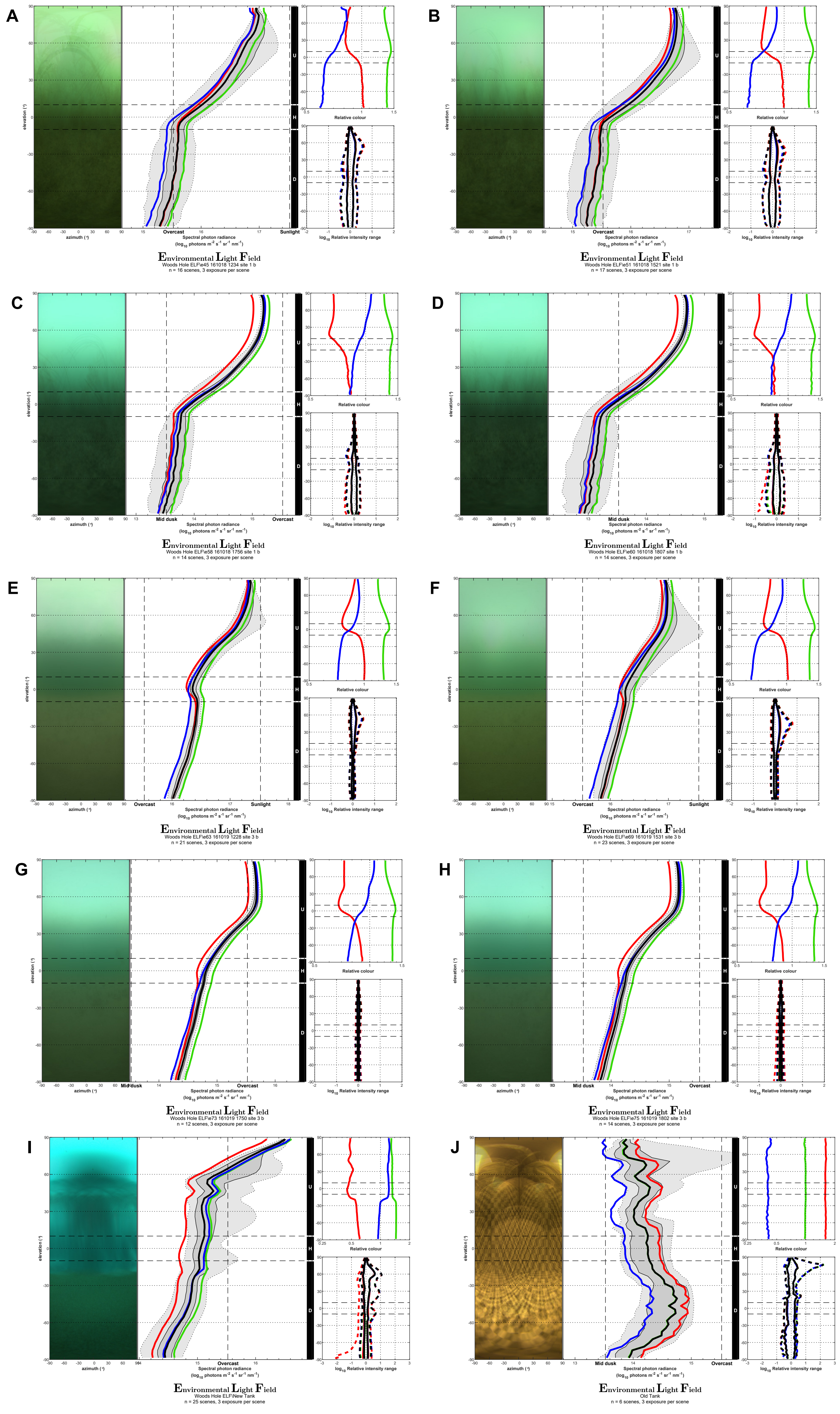

Journal of Experimental Biology • Supplementary information

Fig. S1. (A-J) Environmental light field (ELF) analysis plots of skate habitats and behavioral arenas. The plots were computed and are displayed as described in Nilsson and Smolka (2021). The panels are arranged the same as the averaged scene images in Figure 2E. (A-D) Rock and algae habitat at noon (A), midafternoon (B), before sunset (C), and after sunset (D). (E-H) Sandy habitat at noon (E), midafternoon (F), before sunset (G), and after sunset (H). (I-J) Lab aquaria with optimized (I) and un-optimized lighting (J). The optimized tank was used for behavioral experiments.

Journal of Experimental Biology • Supplementary information
